# Polysaccharides from Passion Fruit Peels: From an Agroindustrial By-Product to a Viable Option for 5-FU-Induced Intestinal Damage

**DOI:** 10.3390/ph16070912

**Published:** 2023-06-21

**Authors:** Karien Sauruk da Silva, Kahlile Youssef Abboud, Carolina Silva Schiebel, Natalia Mulinari Turin de Oliveira, Laryssa Regis Bueno, Lara Luisa Valerio de Mello Braga, Bruna Carla da Silveira, Isabella Wzorek França dos Santos, Everton dos Santos Gomes, Marcelo Biondaro Gois, Lucimara Mach Côrtes Cordeiro, Daniele Maria Ferreira

**Affiliations:** 1 Faculdades Pequeno Príncipe, Programa de Pós-graduação em Biotecnologia Aplicada à Saúde da Criança e do Adolescente, Curitiba 80250-200, Brazil; kariensauruk@outlook.com (K.S.d.S.);; 2 Instituto de Pesquisa Pelé Pequeno Príncipe, Av. Silva Jardim No 1532, Curitiba 80250-200, Brazil; 3 Department of Biochemistry and Molecular Biology, Federal University of Paraná, Curitiba 81531-980, Brazil; kahlilebbd@gmail.com (K.Y.A.);; 4 Programa de Pós-Graduação em Imunologia, Universidade Federal da Bahia, Salvador 40231-300, Brazil; 5 Programa de Pós-Graduação em Biociências e Saúde, Universidade Federal de Rondonópolis, Rondonópolis 78736-900, Brazil

**Keywords:** agroindustrial by-products, agroindustrial residues, *Passiflora edulis*, intestinal damage, intestinal injury, chemotherapy damage, 5-FU

## Abstract

Gastrointestinal mucositis is a serious and dose-limiting toxic side effect of oncologic treatment. Interruption of cancer treatment due to gastrointestinal mucositis leads to a significant decrease in cure rates and consequently to the deterioration of a patient’s quality of life. Natural polysaccharides show a variety of beneficial effects, including a gastroprotective effect. Treatment with soluble dietary fiber (SDF) from yellow passion fruit (Passiflora edulis) biomass residues protected the gastric and intestinal mucosa in models of gastrointestinal injury. In this study, we investigated the protective therapeutic effect of SDF on 5-FU-induced mucositis in male and female mice. Oral treatment of the animals with SDF did not prevent weight loss but reduced the disease activity index and preserved normal intestinal function by alleviating diarrhea and altered gastrointestinal transit. SDF preserved the length of the colon and histological damage caused by 5-FU. SDF significantly restored the oxidative stress and inflammation in the intestine and the enlargement and swelling of the spleen induced by 5-FU. In conclusion, SDF may be a promising adjuvant strategy for the prevention and treatment of intestinal mucositis induced by 5-FU.

## 1. Introduction

The gastrointestinal tract is a complex organ and the largest immunological interface with the environment [1]. At the same time, it plays a fundamental role in the digestion and absorption of nutrients, acts as a protective barrier, and coordinates the difficult task of maintaining the basic characteristics of intestinal homeostasis to various microbial and luminal dietary antigens [2,3]. Exposure to constant chemical, biological, and mechanical stimuli triggers complex immune responses that are finely coordinated by epithelial and non-epithelial cells, which act in an integrated manner to form protective barriers in the mucosa [4]. However, damage to this barrier may occur and disrupt the homeostatic balance. In this case, dysregulated immune responses trigger inflammation, which may be acute or chronic pathological [5,6]. In this sense, various factors can break the protective barrier of the mucosa, such as the antineoplastic drugs used during oncological treatment [7,8,9].

As an antimetabolite chemotherapeutic agent, 5-FU is routinely used alone or in combination to treat various neoplasms such as cervical cancer, esophageal cancer [10], and colon cancer [11]. However, despite its therapeutic efficacy, the use of 5-FU is associated with several serious and complex adverse effects [12,13,14], including inflammation of the intestinal mucosa, also known as intestinal mucositis, which can occur in up to 100% of patients depending on the therapeutic regimen used [15,16]. The disruption of DNA synthesis caused by 5-FU leads to apoptotic cell death [17], especially in tissues with high proliferative potential, as is the case with the crypt cells of the intestinal epithelium [18,19,20]. Damage is also associated with excessive production of reactive oxygen species (ROS), infiltration of neutrophils, and release of proinflammatory cytokines, which contribute critically to the progression and severity of mucositis [21,22]. Importantly, intestinal mucositis is accompanied by nausea, vomiting, diarrhea, and ulcerative lesions that can occur at any site of the gastrointestinal tract [23,24].

Clinical management of intestinal mucositis is based on the patient’s symptoms [25]. Evidence-based guidelines for the management of mucositis have been available since 2004 and are published regularly by the Multinational Association of Supportive Care in Cancer and the International Society of Oral Oncology (MASCC/ISOO) [26]. However, despite continued research, intestinal mucositis remains a significant clinical challenge, especially in severe cases [27,28], and the search for new prevention and/or treatment methods is extremely important.

In this sense, polysaccharides derived from natural products are currently in a remarkable scenario due to the diversity of their biological potential [29,30,31,32,33,34,35,36,37,38,39,40]. Moreover, polysaccharides have been shown to improve the barrier function of the intestinal epithelium in various chemical models of intestinal mucosal inflammation [41,42]. The protective effect might be related to their antioxidant and anti-inflammatory activities [43,44,45,46,47,48,49,50], modulation of immune and inflammatory responses [51,52], direct mucosal protection, regulation of mucins and mucus secretion [41,53,54,55], and alteration of intestinal microbiota [56,57]. Polysaccharides are also abundant in plant by-products such as seeds, bagasse, and peel, which are discarded by the ton during fruit processing and the production of juices and soft drinks [58]. The peels of *Passiflora edulis*, popularly known as yellow passion fruit, which are considered a by-product, are rich in bioactive compounds, including polysaccharides. Tons of peels and seeds are discarded during the processing of yellow passion fruit, accounting for about 50% of the fruit’s weight [59,60]. Passion fruit peel flour ingestion has previously been shown to lower fasting blood glucose in type 2 diabetics [61] and in women with hypercholesterolemia [62]. In animals, improvement in glucose parameters, reduction in serum triglyceride levels, and increased fecal excretion of lipids were observed, which was associated with protection against weight gain [63]. Insulin sensitivity was improved in a high-fat-diet animal model [64], and glucose absorption was slowed in an in vitro upper gastrointestinal tract model [65]. In an animal model of chemically induced ulcerative colitis, supplementation with passion fruit peel flour improved disease parameters, including reductions in diarrhea, rectal bleeding, and weight loss, in addition to reducing inflammation [66,67]. Our research group has previously shown that a fraction of yellow passion fruit peel, rich in polysaccharides (referred to here as SDF), has gastroprotective effects against stomach ulcers [68]. In addition, SDF has been shown to reduce the severity of experimental ulcerative colitis by regulating oxidative stress and inflammation and strengthening the intestinal protective barrier [42].

In view of the above, the aim of our study was to investigate the protective and curative effects of soluble dietary fiber (a polysaccharide fraction, here referred to as SDF) from yellow passion fruit peel in a 5-FU-induced intestinal mucositis model in mice.

## 2. Results

### 2.1. Effect of SDF on Weight Loss, DAI, and Colon Length

#### 2.1.1. Female Mice

**Figure 1:**
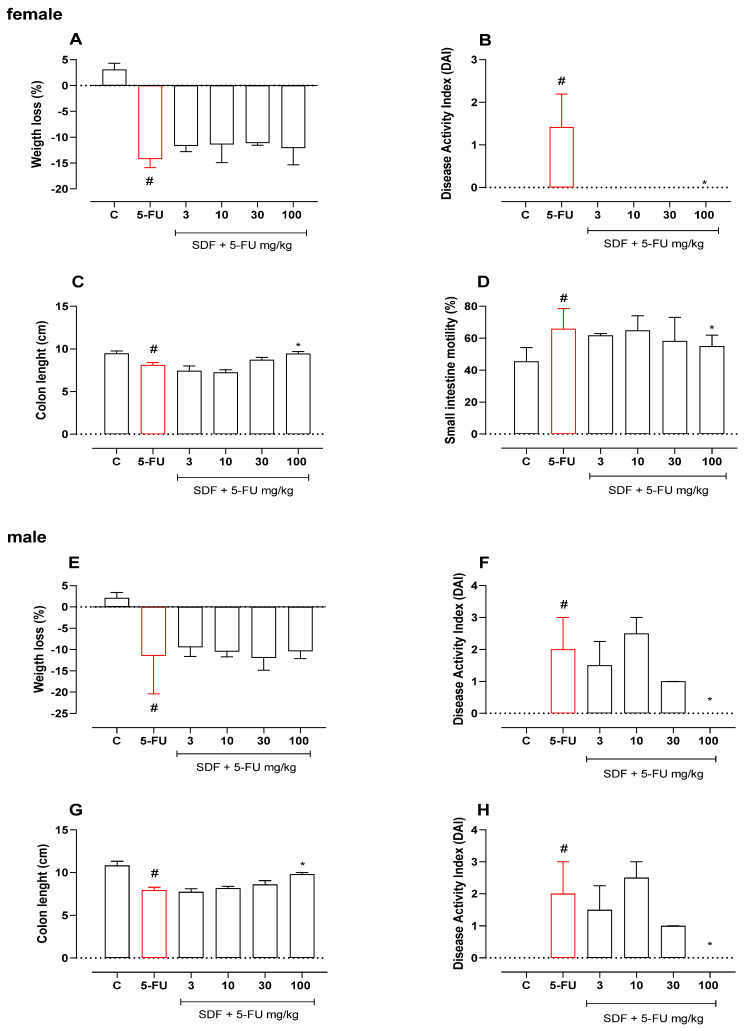
Effect of orally administered SDF on body weight loss, disease activity index, colon length, and intestinal motility in 5-FU-induced intestinal mucositis in female (**A**–**D**) and male (**E**–**H**) mice. * Difference from 5-FU group, # difference from control group to *p* < 0.05.

To analyze the effect of SDF on 5-FU-induced mucositis, parameters indicative of the disease were examined throughout the experimental protocol. On day 10 (three days after 5-FU administration), significant weight loss was observed in the 5-FU group compared with the control group, which persisted until day 12 (14.2%). Treatment with SDF (3, 10, 30, and 100 mg/kg) failed to prevent weight loss compared with the 5-FU group (day 12) (Figure 1A). Although weight loss was not prevented, treatment with SDF (100 mg/kg) prevented the development of DAI (median: 0.00) compared with animals in the 5-FU group (median: 1.0) (Figure 1B). Treatment with 5-FU also reduced the length of the colon (8.10 ± 0.31 cm) compared with the control group (9.45 ± 0.32 cm). On the other hand, treatment with SDF (100 mg/kg) prevented colon shortening by 16% compared with the 5-FU group (Figure 1C).

It is important to note that the anti-mucositis effect was not observed at all doses tested. Only the dose of 100 mg/kg (highest dose tested) promoted the visible beneficial effect. Therefore, we decided to work with this dose in the following experiments. 

#### 2.1.2. Male Mice

As in the experiments with female mice, significant weight loss (median: −11.50) was observed in male mice five days after induction of intestinal mucosal inflammation with 5-FU compared with the control group (median: 2.10). Treatment with SDF (3, 10, 30, and 100 mg/kg) did not prevent weight loss compared with the 5-FU group (Figure 1E).

Although weight loss was not prevented, treatment with SDF (100 mg/kg) significantly prevented the increase in DAI (median: 0.00) compared with 5-FU (median: 2.00) (Figure 1F). In addition, treatment with 5-FU reduced the length of the colon (5-FU: 7.93 ± 0.35 cm) compared with the control group (10.82 ± 0.51 cm). Treatment with SDF (100 mg/kg) prevented 23.70% shortening of the colon (SDF: 9.81 ± 0.18 cm) compared with the 5-FU group (Figure 1G).

Although we did not observe an initial anti-mucositis effect at all doses tested, it is evident that the dose of 100 mg/kg produced a beneficial effect in male mice. Therefore, we decided to work only with the highest evaluated dose (100 mg/kg) in the next experiments.

### 2.2. Effect of SDF on Small Intestinal Motility

#### 2.2.1. Female Mice

Animals in the 5-FU group showed a 30.96% increase in small intestinal motility compared to the control group (median: 45.45%) (Figure 1D). Treatment with SDF (100 mg/kg) prevented the increase in motility (median: 55.00%) compared to the 5-FU group (median: 65.83%).

#### 2.2.2. Male Mice

Male animals with intestinal mucosal inflammation showed no significant difference in small intestinal motility compared with the control group. However, treatment with SDF decreased motility at all doses studied (47.79 ± 3.87, 46.96 ± 4.62, 48.99 ± 2.93, and 42.88 ± 1.21, respectively) compared to the 5-FU group (70.95 ± 2.92%) (Figure 1H).

### 2.3. Effect of SDF on the Weight of Spleen and Liver

#### 2.3.1. Female Mice

**Figure 2:**
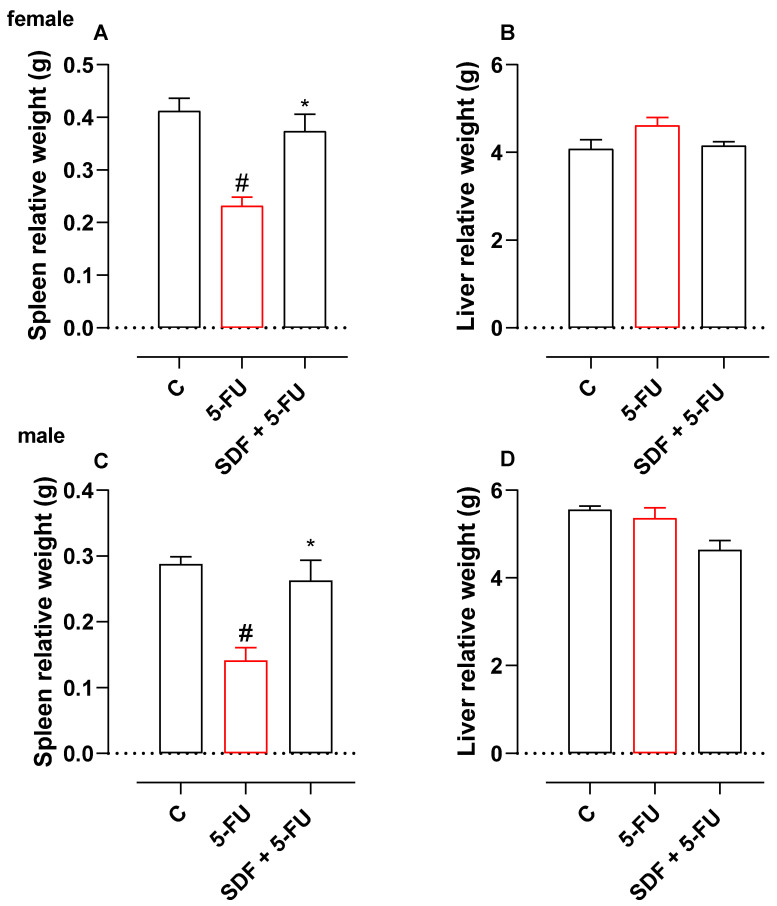
Effect of orally administered SDF on spleen and liver weights in 5-FU-induced intestinal mucositis in female (**A**,**B**) and male (**C**,**D**) mice. * Difference from 5-FU group, # difference from control group to *p* < 0.05.

In animals receiving 5-FU and treated with vehicle alone, spleen weight decreased by 56.09% (0.41 ± 0.02 g) compared with the control group (Figure 2A). However, treatment with SDF (100 mg/kg) prevented the decrease in spleen weight (0.37 ± 0.03 g) compared with the 5-FU group (0.23 ± 0.01 g). No significant difference was observed in liver weight (Figure 2B).

#### 2.3.2. Male Mice

Treatment with 5-FU caused a 50% decrease in spleen weight compared with the control group (0.28 ± 0.01 g). However, treatment with SDF prevented the decrease in spleen weight (0.26 ± 0.03 g) compared with the 5-FU group (0.14 ± 0.01 g) (Figure 2C). No significant difference was observed in liver weight (Figure 2D).

### 2.4. Effect of SDF on Oxidative Stress Parameters

#### 2.4.1. Female Mice

Treatment with 5-FU induced a decrease in duodenal GSH levels (61.52%) and GST activity (68.48%) compared with the control group (359.2 ± 56.32 μg/mg protein and 356.8 ± 25.85 nmol/min/mg protein). SDF prevented GSH depletion and decreased GST activity (293.0 ± 23.36 μg/mg protein and 228.7 ± 30.96 nmol/min/mg protein, respectively) compared with the 5-FU group (138.2 ± 23.76 μg/mg protein and 112.4 ± 17.66 nmol/min/mg protein, respectively).

**Figure 3:**
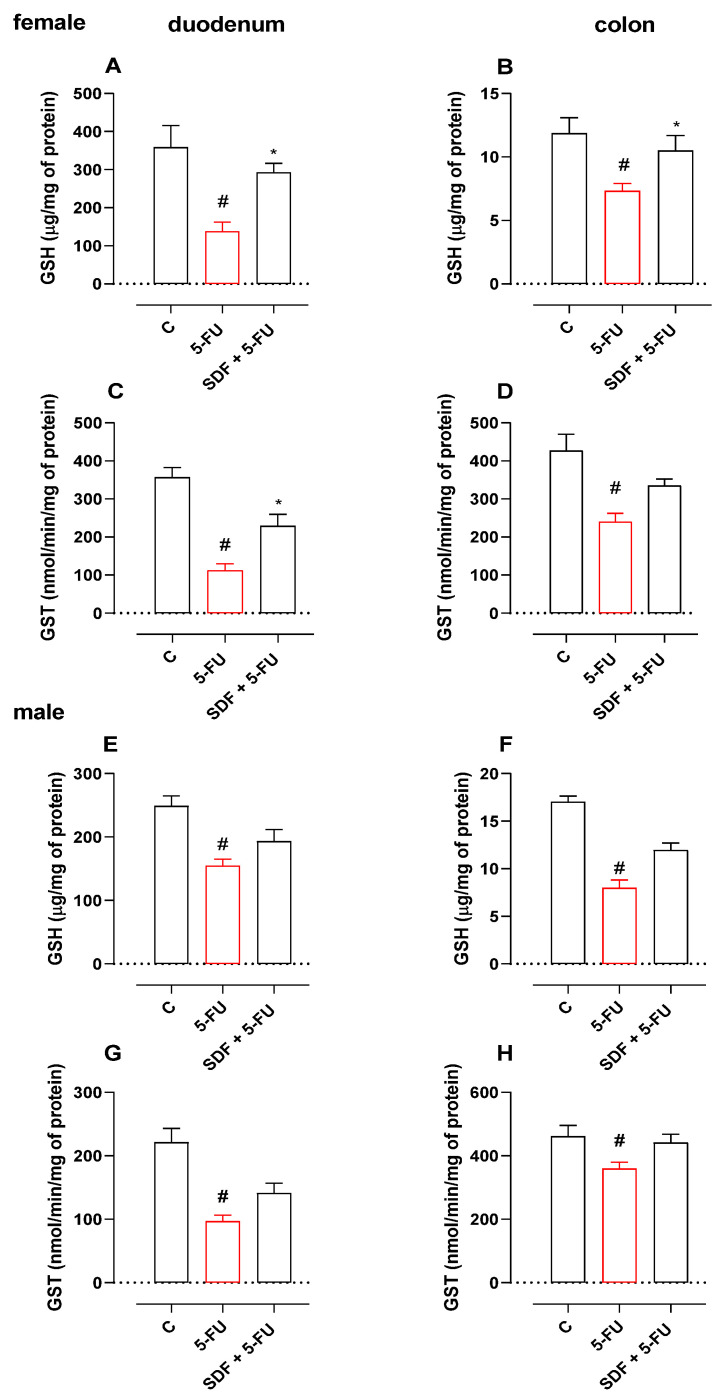
Effect of orally administered SDF on GSH and GST levels in 5-FU-induced intestinal mucositis in female (**A**–**D**) and male (**E**–**H**) mice. * Difference from 5-FU group, # difference from control group to *p* < 0.05.

Treatment with 5-FU induced a decrease in the GSH level (61.75%) and GST activity (56.28%) of colon tissue compared with the control group (11.87 ± 1.21 μg/mg protein and 426.6 ± 43.55 nmol/min/mg protein, respectively). SDF prevented GSH depletion (10.51 ± 1.17 μg/mg protein) but showed no significant difference in GST activity (335.0 ± 17.42 nmol/min/mg protein) compared with the 5-FU group (7.33 ± 0.58 μg/mg protein and 240.1 ± 22.25 nmol/min/mg protein, respectively) (Figure 3A–D).

#### 2.4.2. Male Mice

Treatment with 5-FU decreased duodenal GSH activity and GST levels compared with the control group (248.9 ± 15.94 μg/mg protein and 221.4 ± 21.85 nmol/min/mg protein, respectively). Oral treatment with SDF failed to prevent GSH degradation and GST activity (193.5 ± 18.32 μg/mg protein and 141.5 ± 15.23 nmol/min/mg protein, respectively) compared with the 5-FU group (154.8 ± 10.28 μg/mg protein and 97.02 ± 9.58 nmol/min/mg protein, respectively) (Figure 3E,F).

Treatment with 5-FU induced a 53.11% decrease in GSH levels in colon tissue compared with the control group (17.04 ± 0.60 μg/mg protein). Oral treatment with SDF showed no significant differences in preventing GSH loss (11.98 ± 0.71 μg/mg protein) compared with the 5-FU group (7.99 ± 0.82 μg/mg protein). We found no significant differences in the dosage of GST in colon tissue (Figure 3H).

### 2.5. Effect of SDF on Inflammatory Parameters

#### 2.5.1. Female Mice

**Figure 4:**
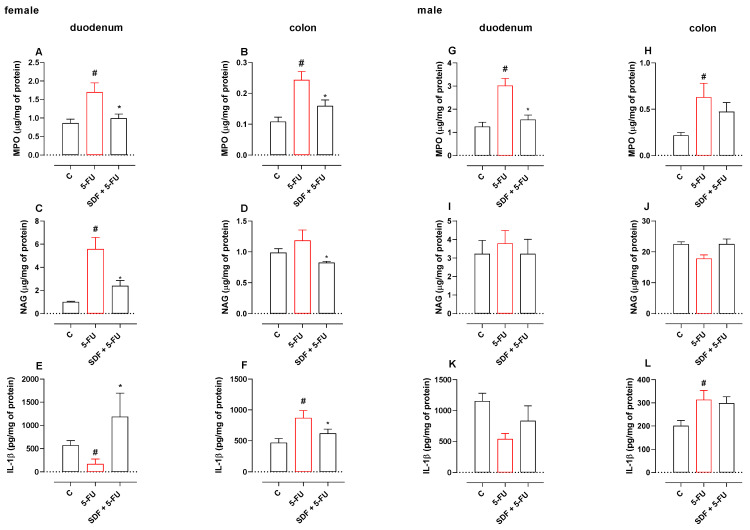
Effect of orally administered SDF on MPO and NAG activities and IL-1β levels in 5-FU-induced intestinal mucositis in female (**A**–**F**) and male (**G**–**L**) mice. * Difference from 5-FU group, # difference from control group to *p* < 0.05.

In the 5-FU group, duodenal MPO and NAG activities were significantly increased by 1.96- and 5.5-fold compared with the corresponding control group (0.86 ± 0.10 μg/mg protein and 1.00 ± 0.05 μg/mg protein, respectively). Treatment with SDF reduced MPO and NAG activities by 41.42% and 57.16% (0.99 ± 0.11 μg/mg protein and 2.39 ± 0.46 μg/mg protein, respectively) compared with the 5-FU group (1.69 ± 0.24 μg/mg protein and 5.58 ± 0.99 μg/mg protein, respectively) (Figure 4A,B).

A 55% increase in MPO activity was observed in the colon tissue of the 5-FU group compared with the corresponding control group (0.10 ± 0.01 μg/mg protein). Treatment with SDF reduced MPO activity by 34% (0.15 ± 0.01 μg/mg) compared with the 5-FU group (0.24 ± 0.02 μg/mg protein).

We observed no significant differences in NAG dosage in the 5-FU group (1.18 ± 0.16 μg/mg protein) for colon tissue compared with the control group (0.98 ± 0.06 μg/mg protein), but SDF at a dosage of 100 mg/kg reduced NAG levels by 30.50% (0.82 ± 0.01 μg/mg) compared with the 5-FU group (Figure 4C,D).

Animals receiving 5-FU had lower levels of IL -1β in the duodenum (171.8 ± 104.9 pg/mg protein) than the control group (569.9 ± 98.69 pg/mg protein). Treatment with SDF increased the IL -1β levels in the duodenum (1188 ± 506.9 pg/mg protein) compared with the 5-FU group.

In addition, it was observed that treatment with 5-FU increased IL -1β levels in the colon tissue (871.5 ± 118.4 pg/mg protein) compared with the control group (468.3 ± 67.87 pg/mg protein). Treatment with SDF reduced IL -1β levels in the colon (619.7 ± 70.29 pg/mg protein) compared with the 5-FU group (Figure 4E,F).

#### 2.5.2. Male mice

Treatment with 5-FU resulted in increased MPO levels in the duodenum and colon (3.01 ± 0.31 μg/mg protein and 0.62 ± 0.15 μg/mg protein, respectively) compared with the control group (1.25 ± 0.18 μg/mg protein and 0.21 ± 0.03 μg/mg protein, respectively). Treatment with SDF did not prevent this increase (1.54 ± 0.20 μg/mg protein and 0.47 ± 0.09 μg/mg protein, respectively). We found no significant differences in the dosage of NAG in the two tissues analyzed (Figure 4G–J).

Under our experimental conditions, animals receiving 5-FU and treated with vehicle had increased levels of IL-1β in the colon (313.5 ± 39.89 pg/mg protein) compared with the control group (201.0 ± 22.97 pg/mg protein). Treatment with SDF was not able to reverse this parameter. Although there is a tendency for change at the other dosages, no significant difference was observed (Figure 4K,L).

### 2.6. Effect of SDF on Duodenum Histological Damage of Female and Male Mice

Sections of duodenum from female and male mice stained with PAS and AB were quantified. Treatment with 5-FU resulted in a decrease in PAS staining (female: 9971 ± 231.2 pixels/field; male: 8089 ± 257.0 pixels/field) compared with the control group (female: 16,098 ± 280.3; male: 21,338 ± 288.5). Treatment with SDF prevented the decrease compared with 5-FU group (female: 16,183 ± 171.5 pixels/field; male: 10,706 ± 175.5 pixels/field).

**Figure 5:**
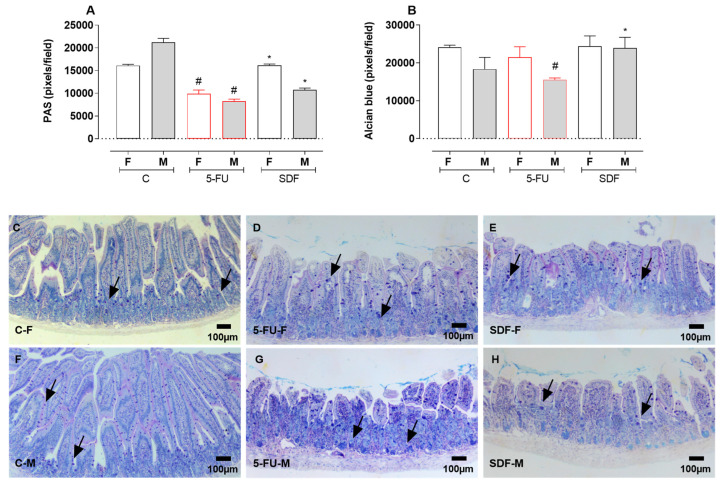
Effect of orally administered SDF on duodenal histological PAS and AB staining of female and male mice. * Difference from 5-FU group, # difference from control group to *p* < 0.05. Images show (**A**) Duodenum stained with PAS (pixel/field); (**B**) Colon stained with Alcian blue (pixel/field); (**C**–**H**) Duodenum stained with PAS-AB (100 µm). C-F: Control group (female), 5-FU-F: 5-FU group (female), SDF-F: SDF group (female), C-M: Control group (male), 5-FU-M: 5-FU group (male), SDF-M: SDF group (male). Arrows: goblet cells.

Treatment with 5-FU induced a decrease in AB staining in male (male: 14,934 ± 479.1 pixels/field) but not in female mice (female: 21,648 ± 696.0 pixels/field) compared with the control group (female: 24,148 ± 526.4 pixels/field; male: 19,760 ± 2135 pixels/field). Treatment with SDF prevented the decrease (male: 25,125 ± 761.9 pixels/field) compared with the 5-FU group (Figure 5A–C).

**Figure 6:**
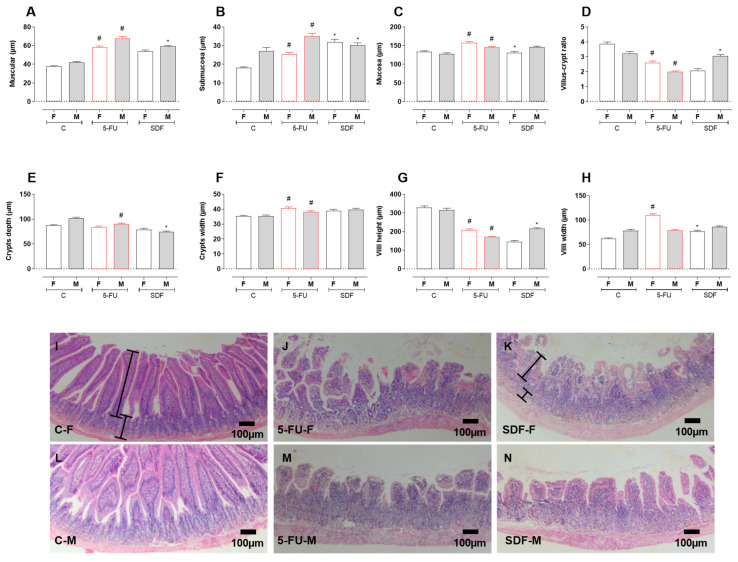
Effect of orally administered SDF on histomorphometry analysis of duodenum in 5-FU—induced intestinal mucosal inflammation in female and male mice (H&E). * Difference from 5-FU group, # difference from control group to *p* < 0.05. Images show (**A**) Muscular (µm); (**B**) Submucosa (µm); (**C**) Mucosa (µm); (**D**) Villus-crypt ratio; (**E**) Crypts depth (µm); (**F**) Crypts width (µm); (**G**) Villus height (µm); (**H**) Villus width (µm); (**I**–**N**) duodenum sections (100 µm) stained with H&E (hematoxylin and eosin). C-F: Control group (female), 5-FU-F: 5-FU group (female), SDF-F: SDF group (female), C-M: Control group (male), 5-FU-M: 5-FU group (male), SDF-M: SDF group (male). Brackets: thickness of the tissue layers (mucosa and submucosa).

Histomorphometric analysis showed atrophy of the muscular, submucosa, and mucosa layers in the 5-FU group (female muscular layer: 58.13 ± 1.49 µm; male muscular layer: 67.74 ± 2.09 µm), (female submucosa layer: 25.41 ± 0.81 µm; male submucosa layer: 35.20 ± 1.33 µm), and (female mucosa layer: 157.8 ± 2.73 µm; male mucosa layer: 145.1 ± 2.42 µm) compared with the control group (female muscle layer: 37.61 ± 0.94 µm; male muscle layer: 41.78 ± 1.13 µm), (female submucosal layer: 18.10 ± 0.49 µm; male submucosal layer: 27.18 ± 1.86 µm), and (female mucosal layer: 133.8 ± 2.32 µm; male mucosal layer: 128.2 ± 2.34 µm). Treatment with SDF improved muscle atrophy in male mice (53.67 ± 1.61 µm) but not in female mice (58.96 ± 1.03 µm), submucosal atrophy in female mice (31.79 ± 1.64 µm) and male mice (30.25 ± 1.26 µm) and prevented mucosal atrophy in female mice (female: 130.4 ± 4.00 µm; male: 146.0 ± 2.50 µm) compared with the 5-FU group (Figure 6A–C).

A decrease in villi and crypts was observed in females and males in the groups receiving 5-FU (female: 2.60 ± 0.10 µm; male: 1.99 ± 0.05 µm) compared with the control group (female: 3.87 ± 0.11 µm; male: 3.22 ± 0.12 µm). SDF prevented the decrease only in the male mice (3.06 ± 0.10 µm) compared to the 5-FU group (Figure 6D). In addition, a decrease in crypt depth was observed in the male 5-FU group (male: 89.98 ± 2.04 µm) compared to the control group (male: 101.8 ± 2.02 µm). SDF treatment improved this parameter (male: 74.17 ± 1.81 µm) compared to the 5-FU group. No statistical differences were observed in females (Figure 6E).

Treatment with 5-FU also resulted in an increase in crypt width (female: 40.62 ± 0.91 µm; males: 38.26 ± 0.66 µm) compared to the control groups (female: 35.28 ± 0.57 µm; male: 35.24 ± 0.83 µm). Treatment with SDF did not prevent the change in crypt (female: 38.80 ± 1.10 µm; male: 39.68 ± 0.89 µm) (Figure 6F). The height of the villi was decreased in the 5-FU group (female: 207.2 ± 6.99 µm; male: 171.2 ± 3.63 µm) compared to the control group (female: 329.2 ± 8.45 µm; male: 315.1 ± 9.75 µm). Treatment with SDF prevented this only in the male mice (female: 144.2 ± 7.65 µm; males: 215.5 ± 4.66 µm) (Figure 6G,I). Villous width was increased only in females of the 5FU group (female: 110.4 ± 2.42 µm; male: 78.87 ± 2.30 µm) compared with the control group (female: 62.12 ± 1.59 µm; male: 78.24 ± 2.86 µm). In female mice treated with SDF, villi width decreased (female: 76.83 ± 2.43 µm; male: 86.12 ± 1.94 µm) compared to the 5-FU group (Figure 6H).

**Figure 7:**
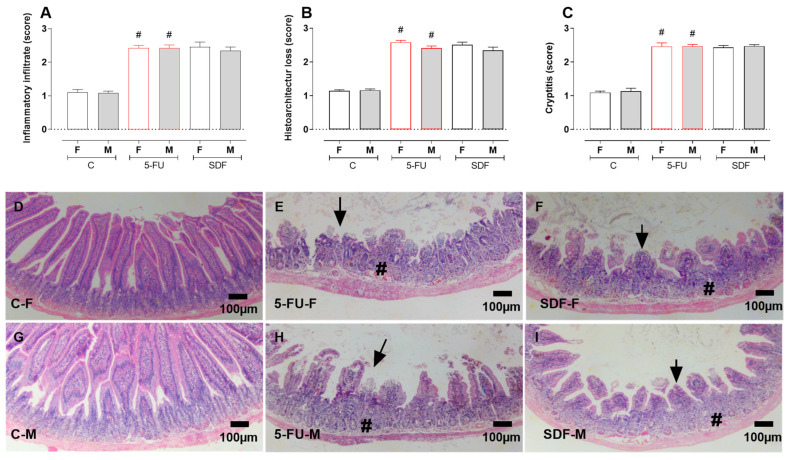
Effect of orally administered SDF on duodenal histopathological analysis in 5-FU -induced intestinal mucosal inflammation in female and male mice (H&E). # difference from control group to *p* < 0.05. Images show (**A**) Inflammatory infiltrate (score); (**B**) loss of histoarchitecture (score); (**C**) Cryptitis (score); (**D**–**I**) duodenum stained with H&E (hematoxylin and eosin) (100 µm). # different from control group to *p* < 0.05. C-F: Control group (female), 5-FU-F: 5-FU group (female), SDF-F: SDF group (female), C-M: Control group (male), 5-FU-M: 5-FU group (male), SDF-M: SDF group (male). Arrows: loss of mucosal architecture, #: inflammatory infiltrate and abscess formation in crypts (cryptitis).

The duodenums of female and male animals receiving 5-FU showed increased infiltration of inflammatory cells (female: 2.42 ± 0.08; male: 2.42 ± 0.09) compared with the control group (female: 1.11 ± 0.07; male: 1.08 ± 0.06), which was not reversed by oral administration of SDF (female: 2.46 ± 0.14; male: 2.35 ± 0.10) (Figure 7A). Histoarchitecture loss was increased in the 5-FU group (female: 2.58 ± 0.06; male: 2.41 ± 0.06) compared with the control group (female: 1.14 ± 0.03; male: 1.16 ± 0.04), which was not reversed by oral administration of SDF (female: 2.51 ± 0.07; male: 2.34 ± 0.10) (Figure 7B). In addition, the crypititis score was increased in the 5-FU group (female: 2.46 ± 0.10; male: 2.47 ± 0.05) compared with the control group (female: 1.09 ± 0.04; male: 1.13 ± 0.08), which was not reversed by oral administration of SDF (female: 2.43 ± 0.05; male: 2.47 ± 0.04) (Figure 7C,D).

**Figure 8:**
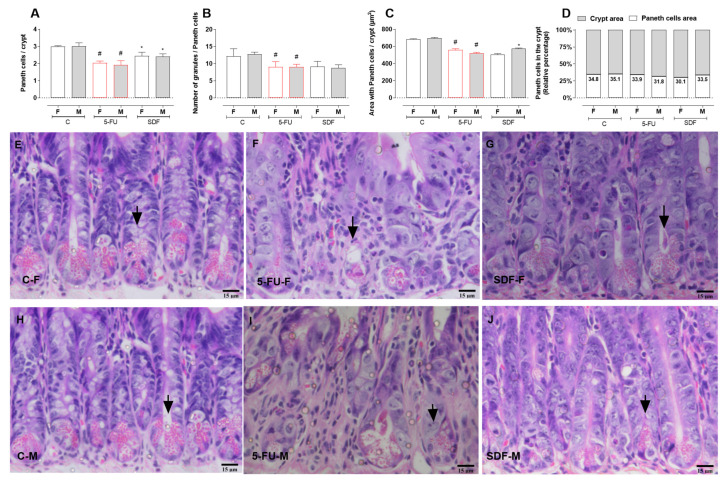
Effect of orally administered SDF on Paneth cells of duodenum in 5-FU-induced intestinal mucosal inflammation in female and male mice (H&E). * Difference from 5-FU group, # difference from control group to *p* < 0.05. Images show: (**A**) Paneth cells/crypt; (**B**) number of granules/Paneth cells; (**C**) area with Paneth cells/crypt (µm2); (**D**) Paneth cells in crypt (relative percentage); (**E**–**J**) small intestine sections stained with H&E (hematoxylin and eosin) (15 µm). C-F: Control group (female), 5-FU-F: 5-FU group (female), SDF-F: SDF group (female), C-M: Control group (male), 5-FU-M: 5-FU group (male), SDF-M: SDF group (male). Arrows: Paneth cells.

Paneth cells of female and male mice receiving 5-FU were decreased (female: 2.05 ± 0.04; male: 1.94 ± 0.05) compared with the control group (female: 2.99 ± 0.04; male: 3.09 ± 0.08). Oral treatment with SDF reversed this decrease (female: 2.49 ± 0.05; male: 2.40 ± 0.06) (Figure 8A). The number of granules per Paneth cell was also decreased in female and male mice receiving 5-FU (female: 9.53 ± 0.55; male: 8.90 ± 0.38) compared with the control group (female: 11.96 ± 0.78; male: 12.74 ± 0.62). Treatment with SDF did not reverse this decrease (female: 9.30 ± 0.55; male: 8.80 ± 0.36) (Figure 8B). The area of the crypt occupied by Paneth cells was also decreased in mice receiving 5-FU (females: 561.2 ± 5.82; males: 520.2 ± 3.71) compared with the control group (female: 683.5 ± 3.77; male: 695.2 ± 10.02). SDF reversed this parameter only in male mice (female: 508.5 ± 4.04; male: 567.9 ± 5.94) (Figure 8C).

Female and male mice in the control group had 34.8 and 35.1% cells per crypt, respectively. Female and male mice in the 5-FU group had 33.9 and 31.8% cells per crypt, respectively. Female and male mice in the SDF group had 30.1 and 33.5% cells per crypt, respectively (Figure 8D,E).

### 2.7. Effect of SDF on Colon Histological Damage of Female and Male Mice

**Figure 9:**
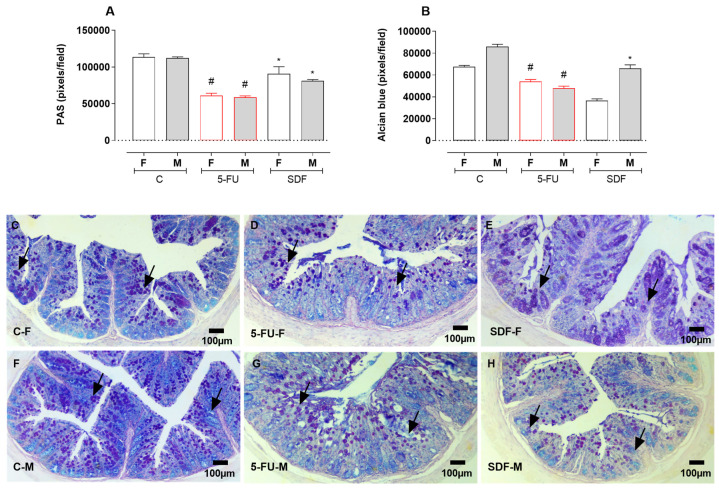
Effect of orally administered SDF on histological analysis of colon goblet cells in 5-FU induced intestinal mucositis in female and male mice (PAS and Alcien Blue). * Difference from 5-FU group, # difference from control group to *p* < 0.05. Images show (**A**) colon stained with PAS (pixel/field); (**B**) colon stained with Alcian blue (pixel/field); (**C**–**H**) colon sections stained with PAS-AB (100 µm). C-F: Control group (female), 5-FU-F: 5-FU group (female), SDF-F: SDF group (female), C-M: Control group (male), 5-FU-M: 5-FU group (male), SDF-M: SDF group (male). Arrows: goblet cells.

Colon sections from female and male mice were stained with PAS and AB and quantified. Treatment with 5-FU resulted in a decrease in PAS staining (female: 61,890 ± 640.3 pixels/field; male: 57,421 ± 1223 pixels/field) compared with the control group (female: 113,415 ± 4623 pixels/field; male: 110,887 ± 1529 pixels/field). Treatment with SDF prevented this reduction (female: 92,099 ± 3486 pixels/field; male: 81,437 ± 1032 pixels/field). Treatment with 5-FU resulted in a reduction in AB staining (female: 54,771 ± 531.4 pixels/field; male: 47,808 ± 872.4 pixels/field) compared with the control group (female: 67,437 ± 1404 pixels/field; male: 85,787 ± 1887 pixels/field). Treatment with SDF prevented this decrease in male but not in female mice (female: 36,998 ± 1830; male: 66,255 ± 1847) (Figure 9A–C).

Histomorphometric analysis showed atrophy of the submucosal layer in the group treated with 5-FU (female: 67.35 ± 2.33 µm; male: 38.76 ± 1.37 µm) compared with the control group (female: 34.14 ± 0.79; male: 25.50 ± 0.64 µm). SDF treatment improved submucosal atrophy (female: 39.92 ± 1.60 µm; male: 45.42 ± 1.71 µm). Although the muscle layer of male mice was improved by SDF treatment (females: 88.16 ± 2.34 µm), no significant difference was observed between the 5-FU and SDF male groups (5-FU females: 90.47 ± 3.22 µm; 5-FU males: 87.61 ± 3.11 µm; SDF males: 111.0 ± 4.58).

**Figure 10:**
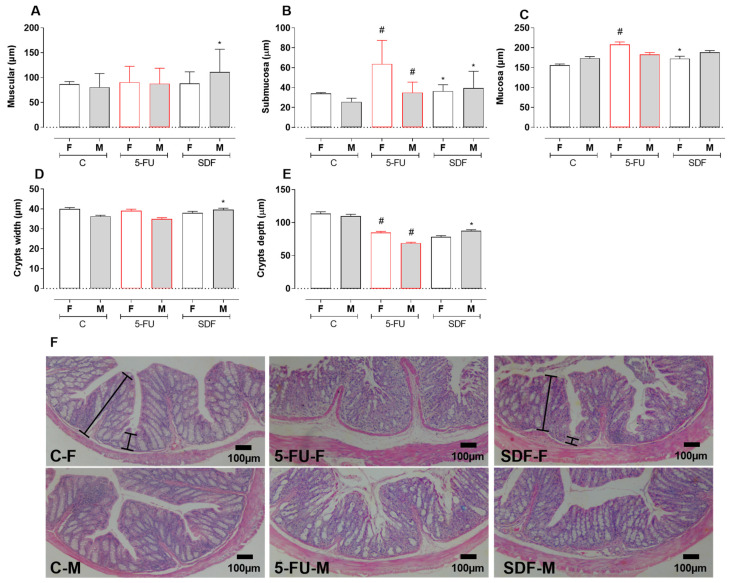
Effect of orally administered SDF on colon histopathological analysis in 5-FU -induced intestinal mucosal inflammation in female and male mice (H&E). * Difference from 5-FU group, # difference from control group to *p* < 0.05. Images show: (**A**) Muscular (µm); (**B**) Submucosa (µm); (**C**) Mucosa (µm); (**D**) Crypts width (µm); (**E**) Crypts depth (µm); (**F**) colon sections stained with H&E (hematoxylin and eosin) (100 µm). C-F: Control group (female), 5-FU-F: 5-FU group (female), SDF-F: SDF group (female), C-M: Con-trol group (male), 5-FU-M: 5-FU group (male), SDF-M: SDF group (male). Brackets: thickness of the tissue layers (mucosa and submucosa).

Treatment with 5-FU induced mucosal atrophy in female mice (208.2 ± 6.27 µm) compared to the control group (155.8 ± 3.49 µm). Treatment with SDF improved this parameter in female mice (172.3 ± 6.39 µm). No significant difference was observed in male mice (Figure 10A–C).

No differences in crypt width were observed in female and male mice receiving 5-FU (female: 39.07 ± 0.82; male: 34.88 ± 0.70) compared with control groups (female: 39.99 ± 0.66; male: 36.27 ± 0.55). SDF treatment (male: 39.58 ± 0.77) showed a significant increase in male mice compared with the 5-FU group (Figure 10D).

In addition, a decrease in crypt depth (female: 84.72 ± 2.03 µm; male: 68.69 ± 1.58 µm) was observed in the 5-FU group compared with the control group (female: 113.6 ± 2.91 µm; male: 109.8 ± 2.78 µm). SDF treatment prevented this decrease in male but not in female mice (female: 78.46 ± 1.68 µm; male: 87.45 ± 1.78 µm) compared to the 5-FU group (Figure 10E,F).

**Figure 11:**
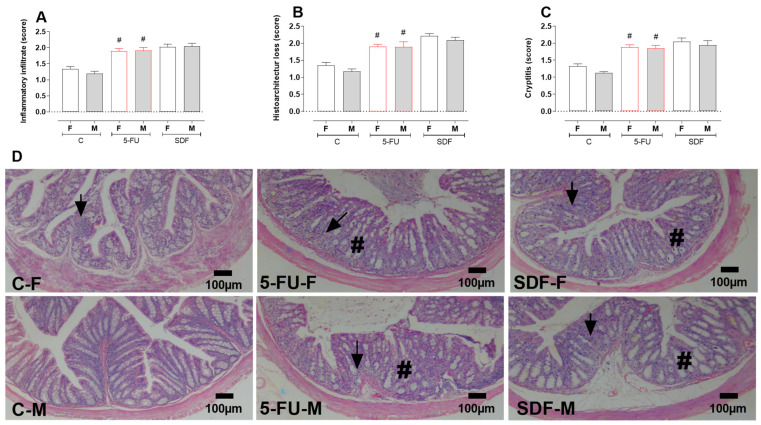
Effect of orally administered SDF on colon histopathological analysis in 5-FU-induced intestinal mucosal inflammation in female and male mice (H&E). # difference from control group to *p* < 0.05. Images show: (**A**) Inflammatory infiltrates (score); (**B**) Histoarchitecture loss (score); (**C**) Cryptitis (score); (**D**) Sections of colon (100 µm) stained with H&E (hematoxylin and eosin). C-F: Control group (female), 5-FU-F: 5-FU group (female), SDF-F: SDF group (female), C-M: Control group (male), 5-FU-M: 5-FU group (male), SDF-M: SDF group (male). Arrows: loss of mucosal architecture, #: inflammatory infiltrate and abscess formation in crypts (cryptitis).

Treatment with 5-FU induced increased infiltration of inflammatory cells (female: 1.89 ± 0.08 µm; male: 1.91 ± 0.09 µm) compared with the control group (female: 1.34 ± 0.07 µm; male: 1.19 ± 0.07 µm), which was not reversed by oral administration of SDF (female: 2.02 ± 0.09 µm; male: 2.05 ± 0.08 µm) (Figure 11A). A loss of histoarchitecture was observed in the 5-FU group (female: 1.90 ± 0.06; male: 1.89 ± 0.15) compared with tfhe control group (female: 1.35 ± 0.08; male: 1.17 ± 0.07), which was not reversed by oral administration of SDF (female: 2.21 ± 0.07; male: 2.09 ± 0.08) (Figure 11B). In addition, the crypititis score was increased in the 5-FU group (female: 1.88 ± 0.07; male: 1.85 ± 0.08) compared with the control group (female: 1.32 ± 0.07; male: 1.13 ± 0.03), which was not reversed by oral administration of SDF (female: 2.05 ± 0.10; male: 1.94 ± 0.13) (Figure 11C,D).

## 3. Discussion

The development of new prevention or treatment strategies with minimal or no adverse effects represents an ongoing effort to control intestinal mucosal inflammation induced by oncologic treatment [24,69,70,71]. In our work, we describe the beneficial effects of oral administration of soluble dietary fiber (SDF) from yellow passion fruit peel in the 5-FU-induced intestinal mucosal inflammation model in female and male mice.

The antimetabolite 5-FU is the most widely used antineoplastic chemotherapeutic agent for the treatment of various solid tumors, including colon and gastric cancers [72,73,74,75]. However, its use leads to the development of serious adverse effects, including gastrointestinal toxicity, routinely described as oral and intestinal mucositis [76]. Intestinal mucositis is a debilitating complication associated with abdominal pain, vomiting, refractory diarrhea, and tissue ulceration [77] and is a consequence of the cytotoxic effect of antineoplastic chemotherapy on the epithelial gastrointestinal layer [78,79]. DNA damage, increased ROS production, cell loss, decreased cell proliferation, and severe inflammation contribute to the damage of the mucosal barrier [80]. Mimicking intestinal mucosal inflammation in rodents, 5-FU (at doses ranging from 50 to 450 mg/kg) is intracellularly converted to active metabolites that interfere with RNA synthesis and the action of thymidylate synthase, leading to cell death [81,82,83]. In animal models used to study the toxic effects of 5-FU, it is still possible to observe weight loss associated with a change in normal intestinal motility followed by diarrhea [81,82,84]. In our study, all the changes described above were observed. Although treatment with SDF did not prevent weight loss, it did prevent the development of DAI and diarrhea. Similar results were observed by Huang et al. [77], who used Glycyrrhiza polysaccharide in DSS-induced ulcerative colitis in mice. The authors showed that Glycyrrhiza polysaccharide could not prevent DSS-induced weight loss. However, DAI was reduced, colon shortening was prevented, intestinal permeability and levels of IL-1, IL-6, and TNF-α were reduced, and levels of IL -10 were increased. A study by Galdino et al. [85], investigating the effects of fructo-oligosaccharide (FOS) pretreatment on 5-FU-induced inflammation of the intestinal mucosa, showed that the polysaccharide tested could not significantly prevent 5-FU-induced weight loss. However, FOS supplementation reduced the inflammatory infiltrate and intestinal permeability, preserved the intestinal mucosa, and increased catalase levels. Pretreatment was still able to maintain acetate and butyrate production at physiological levels.

In addition to dysmotility, 5-FU is known to cause epithelial damage [83], as well as structural and functional damage to the intestinal mucosa [86], which includes severe inflammation, apoptosis, and decreased cellularity of the duodenum and colon [27,87]. Histopathological changes in the duodenum and colon, such as loss of normal architecture, edema, damage to epithelial villi, abnormal structure of the crypt, vacuolization, and inflammatory infiltrates, are also usually reported during the use of 5-FU [88]. In fact, in addition to the above features, we also observed shortening of the colon. Treatment with SDF prevented these changes at the highest dose studied. Similarly, other polysaccharides isolated from natural products have been explored as options for the treatment of inflammation of the intestinal mucosa. For example, oral administration of fucoidan isolated from Acaudina molpadioides at a dose of 50 mg/kg improved the histological architecture of the small intestine in male Balb/C mice after exposure to cyclophosphamide [89]. In addition, an isolated polysaccharide from longan (Dimocarpus longan Lour) promoted intestinal epithelial protection at doses ranging from 100 to 400 mg/kg in the same model of intestinal inflammation [90], indicating the potential of polysaccharides as interesting alternatives for epithelial protection during antineoplastic chemotherapy.

Several mechanisms ensure that the epithelial line is protected from harmful stimuli [91]. In addition to the very important role of the innate immune system, the intestinal epithelium also has a protective mucus layer (the first layer of physical defense of the intestine) that is composed of a complex of mucins and plays an essential role in protecting against digestive enzymes and preventing adhesion and invasion of pathogenic microbes [92,93]. Disruption of its homeostasis, which involves a dynamic balance of production, secretion, expansion, and proteolysis of mucus components [94], can promote dysfunction of this protective barrier and increase susceptibility to lesion development [95]. Indeed, previous studies have shown that 5-FU negatively affects the dynamic mucus barrier [96,97], significantly decreases the number of goblet cells and mucus secretion [98], and negatively affects the expression of mucins [99]. Therefore, therapies that promote mucus retention and increase mucus production and secretion could be interesting strategies for the treatment of chemotherapy-induced intestinal inflammation. Similar to the results of other authors who have shown that polysaccharides improve the mucus barrier [98,99], our results are consistent with the previous statement. Treatment with SDF visibly increased the intensity of staining in the colon of male and female animals.

The exact mechanism by which SDF exerts its beneficial effects is not yet entirely clear. It has already been observed that SDF has a significant gastroprotective effect on gastric ulcer induced in rats [68]. Based on our previous results, we can hypothesize that the protection promoted by SDF may involve the maintenance of normal intestinal motility, preservation of histological architecture, and stimulation of mucus production and secretion. Indeed, polysaccharides isolated from natural products have been shown to increase mucus secretion and mucin expression in models of intestinal inflammation induced by chemotherapeutic agents [54,55,90] and ulcerative colitis [100,101]. In addition, these compounds have been shown to reduce oxidative stress and inflammation. In support of our hypothesis, we have previously shown that SDF (10, 30, or 100 mg/kg, p.o.) prevented body weight loss and colon shortening and improved DAI oxidative stress and inflammatory responses in a model of colitis induced by 5% sodium dextran sulfate (DSS); moreover, SDF improved colon tissue [42].

The formation of reactive oxygen species, followed by a state of drastic oxidative stress, is observed in intestinal tissues after 5-FU application [102,103,104,105,106]. This stage is particularly important because it triggers a series of other events culminating in immunological imbalance and activation of signaling factors such as nuclear transcription factor κB (NF-κB), p53 (phosphoprotein 53), Wnt/β-catenin, caspase-1/3, Bcl-2, and related signaling pathways [107]. Together, these events cause instability in gut barrier homeostasis [108], leading to excessive neutrophil recruitment and infiltration and secretion of a variety of cytokines (e.g., IL -1β and TNF-α), resulting in tissue ulceration and damage [109]. Accordingly, the extent of oxidative stress and inflammatory cell infiltration is directly related to the severity of mucositis. Thus, reducing neutrophil recruitment and maintaining the balance between pro- and anti-inflammatory cytokines is essential for protective immunity and for the prevention of lesions of the intestinal epithelium [110]. Animals treated with 5-FU showed a significant increase in MPO and NAG, indirect markers of infiltration of polymorphonuclear and mononuclear cells (mainly neutrophils and macrophages). We also observed a significant change in IL-1β levels. SDF significantly restored tissue damage, as evidenced by decreased inflammation. In addition, it is worth noting that the enlargement and swelling of the spleen induced by 5-FU, which is normally related to the extent of inflammation of the intestinal mucosa [111], was significantly reduced by SDF. Overall, the present study proved that SDF attenuated the intestinal inflammation induced by 5-FU. The proposed pretreatment can reduce diarrhea, restore normal small intestinal motility, and improve intestinal barrier function.

## 4. Materials and Methods

### 4.1. Soluble Dietary Fiber Extraction and Characterization

The extraction and characterization of soluble dietary fiber (referred to here as the SDF fraction) from passion fruit peels has been described previously [68]. SDF consists of pectic polysaccharide composed of galacturonic acid (92%), with minor amounts of arabinose (3.0%), galactose (2.3%), glucose (1.8%), and trace amounts of rhamnose, mannose, and xylose. NMR and HPSEC analyzes showed that the pectin in the SDF fraction is a highly methyl esterified homogalacturonan (DE = 70%) with a relative Mw of 53 kDa.

### 4.2. Animals and Ethics Statement

Before the start of the experiments, all experimental protocols were submitted to the Ethics Committee for the Use of Animals (CEUA) of the Instituto de Pesquisa Pelé Pequeno Príncipe and approved under the number 047-2019. All persons involved in this project were professionally trained. The procedures were performed in compliance with all requirements, ethical principles, and all relevant requirements and laws. Female and male Balb/C mice (20–30 g, 6–8 weeks old) were obtained from Fundação Oswaldo Cruz (Fiocruz), Paraná, Brazil. Animals were housed in plastic boxes (maximum 12 animals per cage, separate cages for females and males) covered with a layer of wood shavings and environmental enrichment, and were acclimatized at least two weeks before the start of the experiment at controlled temperature (23 ± 2 °C), humidity (60 ± 10%), and lighting (light-dark cycle of 12 h, light at 6 am), with free access to water and food (Nuvilab CR -1, Quimtia S/A, Brazil). The animals were also acclimated to the individuals involved in the development of the experiments. Every two days, the boxes and environmental enrichment were changed and the water and food were replaced. At the beginning of the experiments, animals were randomized, paired by weight, and divided into experimental groups as described below (n = 8–10 animals per group).

### 4.3. 5-FU Intestinal Mucositis Induction

After the acclimation period, animals were randomized, matched by body weight, and divided into smaller groups: (i) control group (water, 0.1 mL/kg, p.o.); (ii) 5-FU group (450 mg/kg of 5-FU [Fauldfluor^®^, Libbs], i.p., and 0.1 mL/kg water, p.o.); and (iii) SDF group (3, 10, 30, or 100 mg/kg SDF p.o. and 450 mg/kg 5-FU, i.p.).

**Figure 12:**
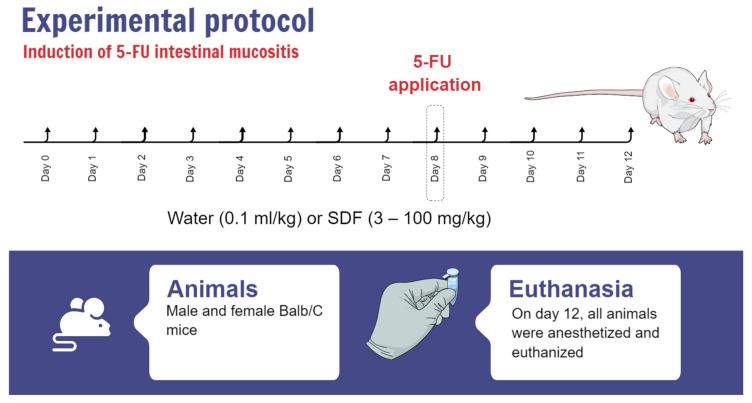
Representative image of the experimental protocol for the induction of intestinal mucosal inflammation.

Animals were treated orally with vehicle (water, 0.1 mL/kg) or SDF (3, 10, 30 or 100 mg/kg) once daily at the same time for 7 consecutive days. On day 8, animals in the 5-FU and SDF groups received a single injection of 5-FU (450 mg/kg (i.p.)). The experimental protocol lasted until day 12, and treatment with vehicle or SDF was maintained until the end of the protocol (Figure 12). On day 12, all animals were anesthetized and euthanized. The duodenum, colon, and spleen were removed and stored in the freezer at −80 °C for further analysis.

Animals were monitored throughout the experimental period for systemic parameters such as weight loss, stool consistency, and presence of blood in the stool. The diarrhea score (disease activity index: DAI), a basic parameter characteristic of the development of intestinal mucosal inflammation in mice, was evaluated using the scoring table proposed by Kurita et al. [112] (Table 1).

### 4.4. Assessment of Intestinal Motility

After a 20-min oral administration of 0.5 mL of a colored marker consisting of 0.05% phenol red diluted in 1.5% carboxymethylcellulose, the animals were euthanized and the length of the small intestine of each animal and the distance traveled by the marker were measured. The data obtained were used to determine intestinal motility.

### 4.5. Tissue Preparation for the Determination of Oxidative Stress and Inflammation Parameters

For determination of oxidative stress and inflammation parameters, the duodenum and colon were homogenized in potassium phosphate buffer solution (PBS, Sigma-Aldrich, P4417, MDL no. MFCD00131855), pH 7.4, and protease inhibitor (Sigma FAST^TM^, Sigma-Aldrich, S8830-20TAB). The homogenate from duodenum and colon was used to measure reduced glutathione (GSH) content. The tissue homogenate was then centrifuged (8900 rpm for 20 min at 4 °C) to obtain the supernatant and pellet of the samples. The supernatant was used to determine glutathione S-transferase (GST) activity, cytokine content (interleukin 1β, [Peprotech, 900-K47]), and protein content. The pellet was resuspended in phosphate buffer containing hexadecyltrimethylamnium (HTAB, Sigma-Aldrich, CAS No. 57-09-0) and used to dose the activities of myeloperoxidase (MPO) and N-acetylglycoside (NAG). Detailed methods are described below.

Protein concentration was determined by the Bradford method [60], using bovine serum albumin (Inlab, CAS No. 9048-46-8) as a standard curve. The results were read in the spectrophotometer at 540 nm.

### 4.6. Oxidative Stress

#### 4.6.1. GSH Determination

GSH was measured according to the method of Sedlak et al. [113]. Aliquots of 50 μL duodenum or colon homogenate were mixed with 50 μL trichloroacetic acid (ATC, 12.5%) and centrifuged at 4 °C and 3000 rpm for 15 min. Then, in a 96-well plate, 10 μL of the supernatant was added to 290 μL TRIS-HCl buffer (400 mM, pH 8.5) (Amresco, CAS No. 1185-53-1) and 10 μL 5,5′-dithiobis-(2-nitrobennomic acid) (DTNB, Sigma-Aldrich, CAS No. 69-78-3) 10 mM. GSH content was then measured in a spectrophotometer at 415 nm (Epoch Microplate Spectrophotometer—BioTek Instruments). The results were interpolated with a standard GSH curve (6.25–400 μg/mL) (Sigma-Aldrich, CAS No. 70-18-8). Results were expressed as μg GSH/mg protein.

#### 4.6.2. GST Determination

GST activity was determined according to the method proposed by Habig et al. [114] with some modifications. The reaction to measure the activity of this enzyme was performed by adding 200 μL of the reaction solution (0.1 M phosphate buffer, 1 mM 1-chloro-2,4-dinitrobenzene CDNB [Sigma-Aldrich, CAS No. 97-00-7], and 1 mM GSH [Sigma-Aldrich, CAS No. 70-18-8]) to 50 μL of the sample supernatant in a 96-well plate. The reaction was then monitored for 180 s in a spectrophotometer (340 nm, Epoch Microplate Spectrophotometer—BioTek Instruments). GST activity was calculated using the extinction coefficient of 9.6/mM/cm for GSH and results were expressed in nmol/min/mg protein.

### 4.7. Inflammatory Parameters

#### 4.7.1. Determination of MPO and NAG Activities

As mentioned previously, MPO and NAG activities were measured in pellets from duodenum and colon samples. For this purpose, the sample pellet was resuspended in 80 mM potassium phosphate buffer (pH 5.4) containing hexadecyltrimethylammonium bromide (HTAB) (Sigma-Aldrich, CAS No. 57-09-0). Briefly, the samples were centrifuged and the supernatant was used to measure MPO and NAG activities.

For MPO measurement, 30 μL of the supernatant was added to 200 μL of a buffer mixture containing phosphate buffer (0.08 and 0.22M) and 0.017% H2O2 (Labsynth, 35% P.A.-A.C.S., CAS No. 7722--84-1) in a 96-well plate. The reaction was started with 18.4 mM TMB (3,3′,5,5′-tetramethylbenzidine, Sigma-Aldrich, CAS No. 207738-08-7) and incubated at 37 °C for 3 min. The reaction was stopped with 30 μL sodium acetate (Quimibras, A.C.S., CAS No. 127-09-3) and read in a spectrophotometer at 620 nm (Epoch Microplate Spectrophotometer—BioTek Instruments). Results were expressed as μg MPO/mg protein.

For the determination of NAG, 25 μL of supernatant was added to 25 μL NAG solution (4-nitrophenyl-N-acetyl-β-D-glucosaminide, Sigma-Aldrich, CAS No. 3459-18-5) and 100 μL citrate buffer (50 Mm, pH 4.5, Synth, CAS No. 6132-04-3). The reaction was incubated at 37 °C for 60 min, stopped with 100 μL glycine buffer (200 Mm, pH 10.4, Sigma-Aldrich, CAS No. 207300-76-3), and read in a spectrophotometer at 405 nm (Epoch Microplate Spectrophotometer—BioTek Instruments).

#### 4.7.2. Evaluation of IL-1β

Il-1β levels were determined by enzyme-linked immunosorbent assay (ELISA) according to the manufacturer’s recommendations (Peprotech^®^). Absorbance was measured using a spectrophotometer (Epoch Microplate Spectrophotometer—BioTek Instruments, Charlotte, VT, USA). Results were interpolated with a standard linear curve for each cytokine and expressed in pg/mg protein of each sample.

### 4.8. Histopathological Analysis

After the experimental protocol for inflammation of the intestinal mucosa was developed, samples were taken from the duodenum and colon to study the histopathological changes. For this purpose, the collected tissues were incubated in buffered formalin (Neon, CAS no. 50-00-0) for 16 h. They were then transferred to ethanol and ether solutions (Abba Quimica, CAS No. 64-17-5 and CAS No. 60-29-7). For preparation of histology blocks, specimens were embedded in paraffin wax (Reagen, CAS No. 8002-74-2). Histology slides were prepared with 5 µm thick sections (rotary microtome, American Optical, 820–54637). All slides were stained with H&E, Periodic Acid Schiff, and Alcian Blue (PAS + AB). For histopathological analysis, images of the slides were captured using a digital camera (Olympus^®^ CX43RF, 3.0 megapixel) connected to an optical microscope (Olympus^®^ EP50).

### 4.9. Histomorphometric Analysis

H&E-stained sections were used to measure the thickness of the muscular, submucosa, and mucosa layers and the depth and width of the crypts in the duodenum and colon. Height and width of villi were measured in the duodenum, with width determined by the average of three measurements at the base, middle third, and tip of each villus. The ratio of villi to crypts was determined by dividing villus height by crypt height. One hundred measurements were made for each parameter studied, with 10 measurements per parameter for each mouse and from 10 mice per group. For this purpose, 16 images (4/quadrant/mouse from 10 mice per group) were acquired with a high-resolution camera (Leipzig, HI -speed, 2.0 megapixels), connected to a light microscope (Leipzig Solstice 5Xi eLED) with 10× objectives, and transferred to a microcomputer. Measurements were performed using Image-Pro Plus software (Media Cybernetics, Rockville, MD, USA) [115,116].

### 4.10. Quantification of Goblet Cells

Sections stained with Periodic Acid-Schiff (neutral mucin-like glycoproteins) and Alcian Blue (acidic mucin) were used to quantify goblet cells producing and releasing neutral and acidic mucins, respectively. For mucin quantification, 10 images per mouse from 10 mice per group were acquired with a 10× objective and a high-resolution camera (Leipzig, HI -speed, 2.0 megapixel) connected to the light microscope (Leipzig Solstice 5Xi eLED) and transferred to a microcomputer. ImageJ^®^ software version 1.53k (Schneider et al., 2012) [117] was used to quantify the mucins. The results were expressed in pixels per microscopic field [116].

### 4.11. Quantitative and Histomorphometric Analysis of Paneth Cells

H&E-stained sections were used to quantify and measure Paneth cells. Paneth cells in 64 intestinal crypts of each mouse were quantified, in 10 mice per group. The eosinophil granules present in 10 Paneth cells of each mouse from 10 mice per group were quantified. The profile of the area occupied by Paneth cells in 64 intestinal crypts of each mouse was measured in 10 mice per group. For this purpose, 20 images (five images/quadrant/mouse of 10 mice per group) were acquired with a high-resolution camera (Leipzig, HI -speed, 2.0 megapixel) coupled to a light microscope (Leipzig Solstice 5Xi eLED) with objectives of 40× or 100× and transferred to a microcomputer. The 100× images were used to quantify the eosinophil granules of Paneth cells. Quantitative and histomorphometric analyzes were performed using Image-Pro Plus software (Media Cybernetics, Rockville, MD, USA) [115,118].

### 4.12. Histopathological Evaluation

H&E-stained sections were used for histopathologic evaluation of the intestinal wall of the duodenum and colon of mice. Histopathological findings were classified into three categories in accordance with previous studies: (i) presence and distribution of inflammatory infiltrates in the submucosa or intestinal mucosa; (ii) loss of histoarchitecture of the intestinal mucosa due to flattening of the mucosa (or villi in the duodenum), depletion of goblet cells, epithelial erosion, ulceration, or abscess formation; and (iii) inflammatory infiltrates in the intestinal crypts (cryptitis). Histopathologic findings were graded from 0 to 3 according to severity, with 0 being normal and 3 being marked. Ten microscopic fields from each mouse (of 10 mice per group) were examined blindly with a light microscope (Leipzig Solstice 5Xi eLED) at a 40× objective (and 100× if necessary to confirm structures) [42,115].

### 4.13. Statistical Analysis

All data were subjected to a normality test before the statistical test was performed. Parametric data were expressed as mean values ± S.E.M. Data that did not have a normal distribution were expressed as median and interquartile range. Difference between groups was determined by analysis of variance ANOVA (one-way ANOVA or two-way ANOVA), followed by Bonferroni test, or Kruskal–Wallis test, followed by Dunn test (depending on data characteristics). Differences with *p* < 0.05 were considered statistically significant.

## 5. Conclusions

Finally, we present here results highlighting the potential of agroindustrial waste products, especially yellow passion fruit peels. Although the mechanism of action of SDF needs to be better explored, its beneficial effects on intestinal mucositis have been demonstrated. Thus, we can conclude that the polysaccharides extracted from the agroindustrial by-product of yellow passion fruit (*Passiflora edulis*) peel could be an interesting option for the treatment of 5-FU-induced intestinal inflammation and that agroindustrial waste products need to be valued for the use and incorporation of their bioactive compounds into products for health benefits.

## Figures and Tables

**Table 1:** Score for the evaluation of feces consistency.

Feces Appearance	Score
normal	0
slightly altered or damp	1
moist with little perianal dirt	2
moist with perianal dirt	3

## Data Availability

Data will be made available on request.

## Artwork

Figure created by Daniele Maria-Ferreira in the Mind the Graph platform paid account (www.mindthegraph.com).
